# The Treatment of Pneumonia

**Published:** 1893-08

**Authors:** 


### THE TREATMENT OF PNEUMONIA.

Dr. H. A. Hare (*Medical World*) bases the treatment of pneumonia upon a recognition of the general condition of the patient rather than upon the cause of his illness. If the patient has been strong and healthy until attacked and is seen within a short time after the initial chill and the consequent rise in bodily temperature, it will be found that his pulse is full and bounding, the cheeks flushed and the respiration somewhat rapid. At this time, and at no other, is aconite, veratrum viride, or antimony indicated, and they should be given fearlessly while they are given. From two to three drops of the tincture of veratum viride or a similar quantity of aconite, preferably the former, are given every half hour until the decrease in pulse rate and pulse force and the appearance of a slight perspiration indicate that the full influence of the drug has been exerted. If veratrum viride has been given early enough it is certainly competent to abort an acute croupous pneumonia occurring in a previously healthy individual. If it is not given early enough the result of its administrrtion will be to hurt the patient. If there is marked dullness on percussion, with blowing breathing and the typical, fine crepitant rale of pneumonia, the use of these cardiac sedatives is contraindicated in most cases, because the disease has become thoroughly instituted and the stage is past when the remedy can do good. Where there is tendency to cardiac failure and the apex beat of the heart seems at all muffled or feeble, digitalis is indicated. The best results from its administration will follow its use in the dose of not less than ten minims every eight hours rather that smaller doses frequently repeated. Particularly is digitalis indicated if the pulse seems feeble. Where the skin has a tendency to be moist and the pulse, although large in volume, from the action of digitalis, seems to be surrounded by a relaxed arterial wall, the tincture of belladonna in the dose of five drops every four hours during the night and day will produce a wonderful change for the better. In some instances where there is considerable dyspnœa, it is well to administer no less than 1-40 and frequently 1-20 of a grain of strychnine every four to eight hours with the digitalis, and if the cough persists in the face of the belladonna, 1-12 to 1-8 of a grain of morphine may be given hypodermically. When the crisis takes place in pneumonia we should always be on the lookout for collapse and cardiac failure, and for the treatment of this accident no remedies are equal to full doses of strychnine and atropine. In the asthenic cases of pneumonia the use of digitalis, belladonna and strychnine must be resorted to almost from the very first. Wet cups or leeches do much good in early stages of acute sthenic pneumonia, but dry cups should be employed in the early stages of asthenic pneumonia.

